# Supplementary material for: Unveiling the structural arrangements responsible for the atomic dynamics in metallic glasses during physical aging
Source: Nat Commun. 2016 Jan 20;7:10344. doi: 10.1038/ncomms10344 (PMC4735801; doi:10.1038/ncomms10344)
Supplement: Supplementary Information — Supplementary Figures 1-5, Supplementary Notes 1-3 and Supplementary References [file ncomms10344-s1.pdf]

Supplementary Information

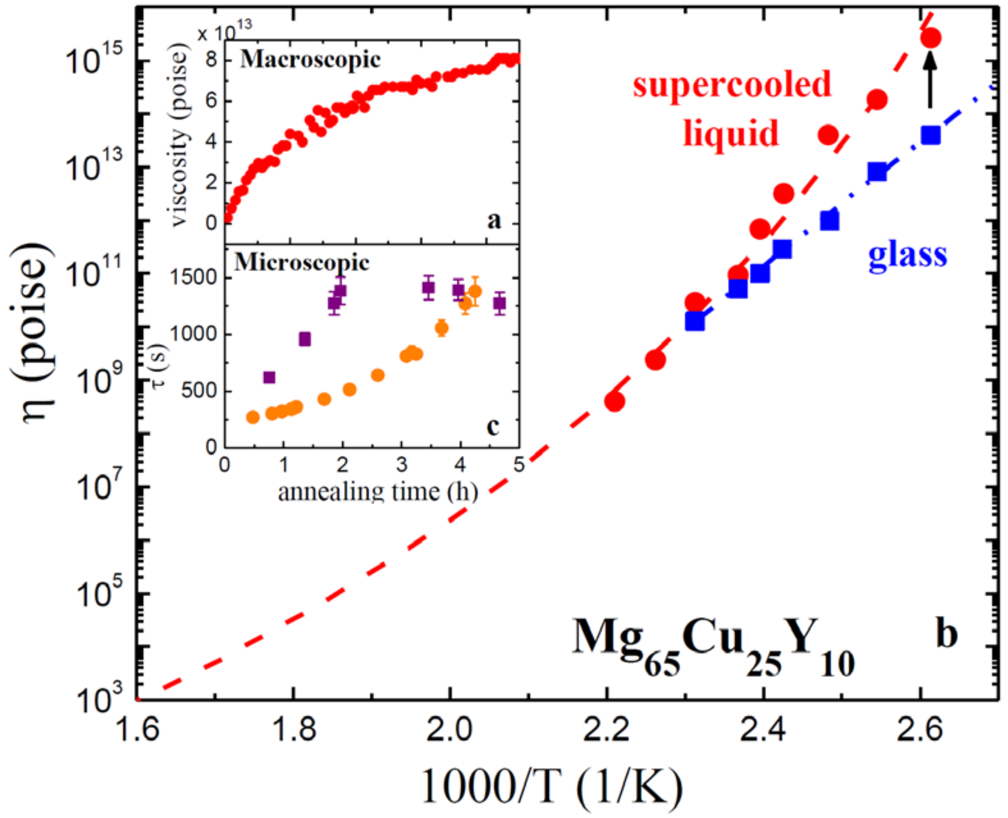

Supplementary Figure 1: (a) Viscosity data measured after a long annealing at 403 K. Data taken from [2]; (b) Arrhenius plot of viscosity data measured in the glass (blue squares) and the supercooled liquid (red circles). The arrow indicates the direction of the aging. Adapted from [2]; (c) relaxation time measured with XPCS in two as-quenched glasses, one heated with isothermal steps (orange circles) and another one pre-annealed close to Tg (purple squares). Data taken from [3].

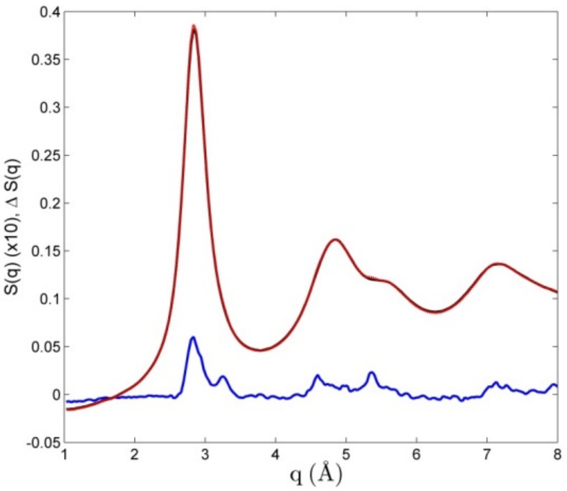

Supplementary Figure 2: S(q) measured at the beginning (black) of the isotherm at 513 K and after 2h (red) is reported dividing its intensity by a factor of 10. In blue the difference between the two curves shows distinct crystalline peaks.

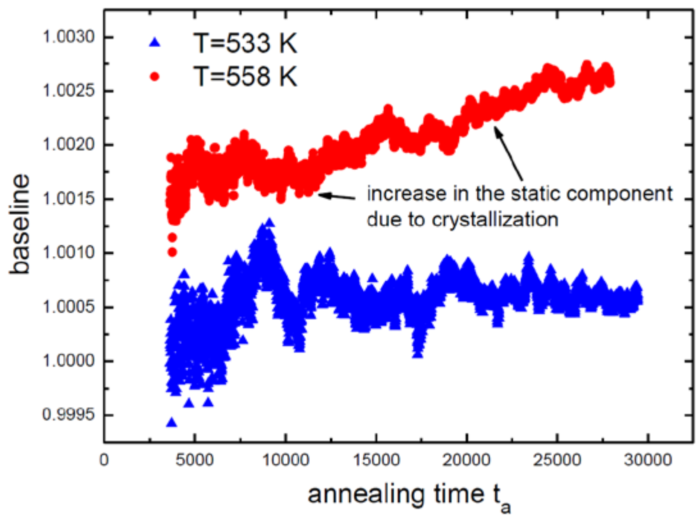

Supplementary Figure 3: Baseline obtained from the analysis of XPCS data taken during long annealing at T=533 K (blue triangles) and T=558 K (red circles). The baseline starts to increase at 558 K due to a larger presence of static contribution in the speckles patterns signs of the concomitant crystalline growth.

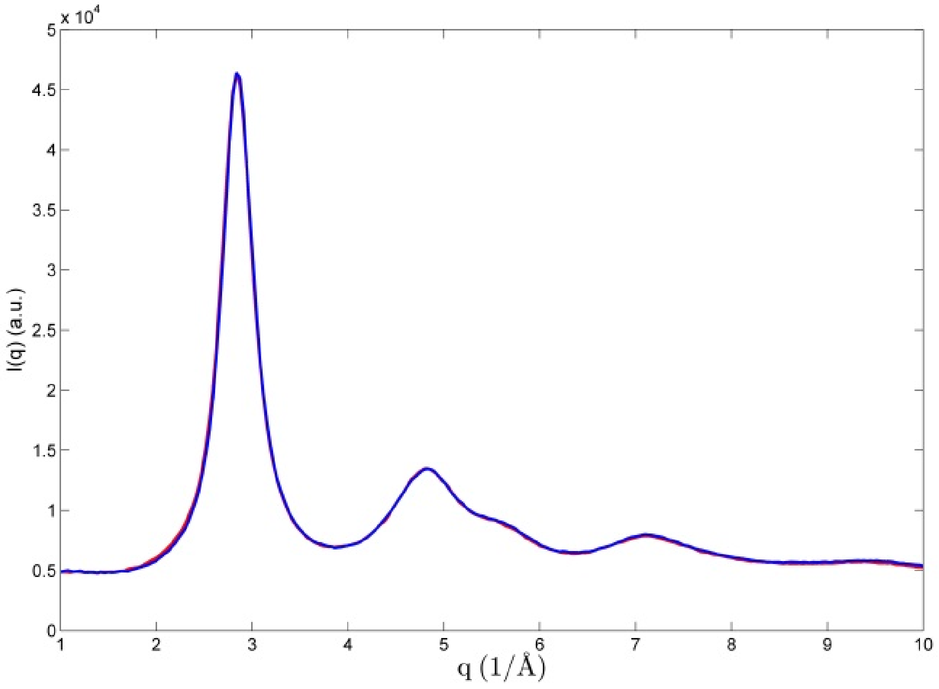

Supplementary Figure 4: Difference in the diffraction pattern integrated parallel and perpendicularly to the ribbon axis in our experiment, at the end of the isotherm at T=160C. Blue line: perpendicular to the ribbon axis, Red line: parallel to the ribbon axis.

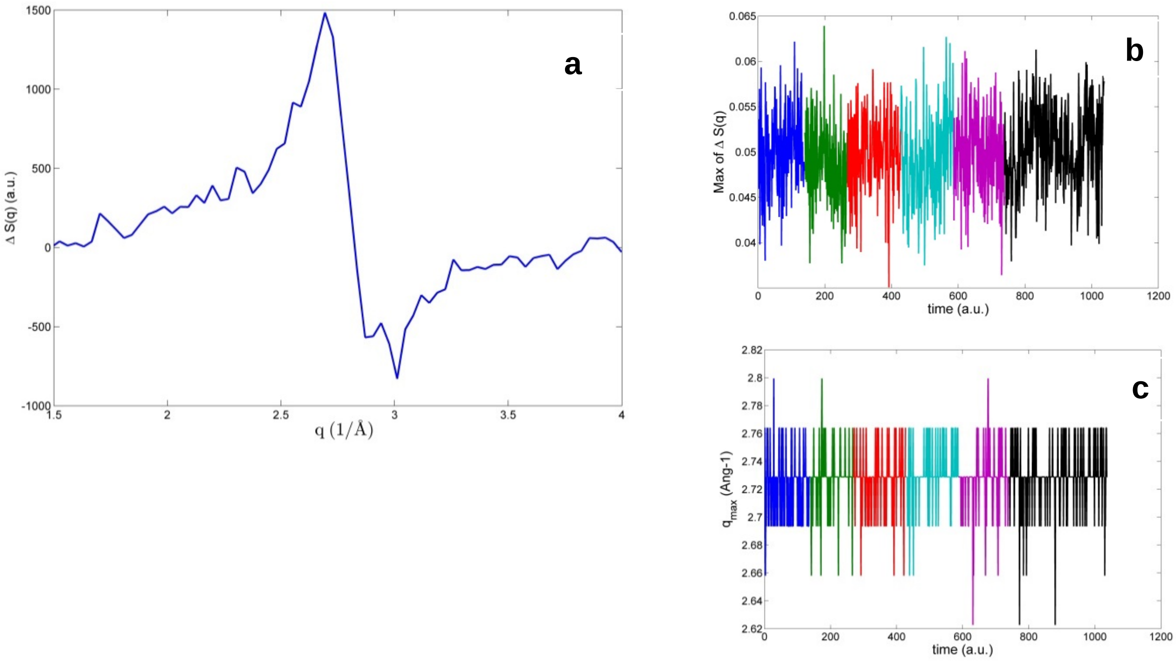

Supplementary Figure 5: (a): ΔS(q)=S(parallel)-S(perpendicular) at the end of the isotherm at T=160C. (b) and (c): evolution with time and temperature of the intensity and position of the maximum of ΔS(q). The color code signaling the different isotherms is the same as in the article.

Supplementary Notes

Supplementary Note 1

Aging in metallic glasses. Physical aging in polymeric, metallic or more conventional inorganic glasses is commonly studied by looking at the temporal evolution of a given observable, keeping fixed other external parameters, such as the temperature and the pressure of the system. Macroscopic quantities like viscosity or elastic constants usually evolve with annealing time in a way which depends on the previous thermal history and which can often be described accurately by stretched exponential functions [1]. Differently, the microscopic aging recently observed in rapidly quenched metallic glasses at the atomic level is characterized by a hierarchy of completely distinct behaviours which contrast from the continuous macroscopic evolution. This is shown in Supplementary Figure 1 for a Mg-based metallic glass. Panel (a) shows viscosity data measured by Busch et al. [2] during a long annealing at 403 K. At short times the system is in the out-of-equilibrium glassy state and then the viscosity increases continuously with time toward the equilibrium value in the corresponding supercooled liquid.
This evolution from the glass to the viscous liquid is shown in panel (b) for data taken for distinct temperatures at short (blue squares) and long (red circles) annealing times. Panel (c) shows the structural relaxation time measured by Ruta et al. [3] with XPCS during annealing at 403 K but following two distinct thermal protocols. Differently from the viscosity data (panel (a)), τ exhibits a fast exponential aging which can be followed by a stationary regime.

Supplementary Note 2

Crystallization. Starting from T=493K we observe a discontinuous reduction of the initial plateau of the density-density correlation function, which corresponds to the thermal

activation of a faster secondary relaxation process. This secondary relaxation process appears simultaneously with a sharper narrowing of the FSDP, indicative of an increased strength of the ordering mechanism in the medium range. This suggests that the secondary relaxation process is a precursor to crystallization, likely related to the appearance and disappearance of nuclei during nucleation.

While at 493K we cannot detect any crystalline peak in our XRD spectra, we do detect an incipient crystallization at 513 K. We report in Supplementary Figure 2 the S(q) at the beginning of the isotherm at 513K and after 2 h, and the difference between them. Crystalline peaks are clearly visible. The corresponding crystalline volume fraction is as small as 0.5%, as estimated from the missing intensity in the amorphous signal once the diffracted intensity has been renormalized by the incident flux.

In another experiment, which is not the object of this article, we have found that at this temperature crystallization proceeds very slowly, so that after 7h the crystalline volume fraction is still only 0.8%.

The detected incipient crystallization strongly supports our interpretation of the fast secondary relaxation as related to nucleation or phase-separation and crystallization.

From the dynamical point of view, the crystallization would give raise to an increase in the static component in the speckles patterns measured with XPCS due to the growth of the crystalline peaks. However this effect becomes visible only for much higher crystalline fractions than those above reported. This is shown in Supplementary Figure 3 where we compare the baseline obtained from the analysis of XPCS data taken at 533K and 558K, thus at temperatures higher than those in Supplementary Figure 2 and in our article. While at 533K the baseline is still constant and ~1, signs of a dominant amorphous matrix, it jumps to a higher value upon increasing T, for then continuously increase with annealing time, likely due to the activation of a steady growth of the crystalline component of the spectra.

Supplementary Note 3

Structural anisotropy. It is quite renowned that MG may display structural anisotropy when subject to anisotropic stress, like uni-axial tension or compression, but also due to the production processing. In presence of such anisotropy, the shift of the FSDP can be related to an anelastic strain due to bond-orientational anisotropy [4,5].

In our experiment no stress is applied, thus we can safely rule out the existence of a stress-induced anisotropy. However it is still possible that a bond-orientational anisotropy has been induced during the melt-spinning quench.

As reported in Ref. [5] the difference between the S(q) measured perpendicularly and parallel to the ribbon axis for a fast-quench induced anisotropy can be dramatic. Such difference is localized at large q, $q>4.5\ A^{-1}$, related to the short range. Supplementary Figure 4 reports the integration of the bidimensional signal in a 5 degrees wide slice perpendicular and parallel to the ribbon axis in our experiment. No significant difference can be appreciated.

In order to emphasize any difference, we compute the difference between parallel and perpendicular configuration, reported in Supplementary Figure 5 (a): a difference is indeed present, meaning that there is actually a residual uniform strain in our sample. However, it does not evolve with time or temperature (Supplementary Figure 5 (b) and (c)).

It is thus clear that although some anisotropy exists in our spectra, it does not evolve during the whole experiment, thus it is not related to the observed aging and relaxation processes.
The constancy of the intensity and position of $\Delta S(q)$ insures that all our findings are independent from it and hold true.

Supplementary References

[1] Wang, W. H. The elastic properties, elastic models and elastic perspectives of metallic glasses. Prog. Mater. Sci. **57**, 487{656 (2012).

[2] Busch, R., Liu, W. & Johnson, W. L. Thermodynamics and kinetics of the mg65cu25y10 bulk metallic glass forming liquid. J. Appl. Phys. **83**, 4134{4141 (1998).

[3] Ruta, B. *et al.* Atomic-scale relaxation dynamics and aging in a metallic glass probed by x-ray photon correlation spectroscopy. Phys. Rev. Lett. **109**, 165701 (2012).

[4] Suzuki, Y., Haimovich, J. & Egami, T., Bond-orientational anisotropy in metallic glasses observed by x-ray diffraction, Phys. Rev. B 35, 2162 (1987)

[5] C.G. Windsor, D.S. Boudreaux & M.C. Narasimhan, A search for anisotropy in the structure factor of amorphous Pd0.8Si0.2 ribbon, Phys. Lett. 67A, 282 (1978)
